# mPILOT-Magnetic Field Strength Based Pedestrian Indoor Localization

**DOI:** 10.3390/s18072283

**Published:** 2018-07-14

**Authors:** Imran Ashraf, Soojung Hur, Yongwan Park

**Affiliations:** Department of Information and Communication Engineering, Yeungnam University, Gyeongsan, Gyeongbuk 38541, Korea; ashrafimran@live.com (I.A.); sjheo@ynu.ac.kr (S.H.)

**Keywords:** indoor localization, deep learning, smartphone sensors, geomagnetism, pattern matching, fingerprinting, pedestrian dead reckoning

## Abstract

An indoor localization system based on off-the-shelf smartphone sensors is presented which employs the magnetometer to find user location. Further assisted by the accelerometer and gyroscope, the proposed system is able to locate the user without any prior knowledge of user initial position. The system exploits the fingerprint database approach for localization. Traditional fingerprinting technology stores data intensity values in database such as RSSI (Received Signal Strength Indicator) values in the case of WiFi fingerprinting and magnetic flux intensity values in the case of geomagnetic fingerprinting. The down side is the need to update the database periodically and device heterogeneity. We solve this problem by using the fingerprint database of patterns formed by magnetic flux intensity values. The pattern matching approach solves the problem of device heterogeneity and the algorithm’s performance with Samsung Galaxy S8 and LG G6 is comparable. A deep learning based artificial neural network is adopted to identify the user state of walking and stationary and its accuracy is 95%. The localization is totally infrastructure independent and does not require any other technology to constraint the search space. The experiments are performed to determine the accuracy in three buildings of Yeungnam University, Republic of Korea with different path lengths and path geometry. The results demonstrate that the error is 2–3 m for 50 percentile with various buildings. Even though many locations in the same building exhibit very similar magnetic attitude, the algorithm achieves an accuracy of 4 m for 75 percentile irrespective of the device used for localization.

## 1. Introduction

The Global Positioning System (GPS) serves as the de facto technology due to its precisely accurate positioning information outdoor. However, GPS inability to work in the indoor environments necessitated the researchers to delve into other alternatives for indoor positioning. Indoor localization got a huge interest, especially during the last decade, primarily due to the excessive use of smartphones. In addition, various companies’ services, which are collectively called Location Based Services (LBS), also accelerated this endeavor as indoor localization serves as the backbone of these services. Not only that, such indoor localization can be extremely helpful to locate workers working in emergency situations such as fire, earthquake, etc. Indoor positioning technology can be broadly divided into two categories: infrastructure dependent systems and infrastructure independent systems. The former either makes the use of existing devices such as WiFi [1,2,3] or installs new hardware such as placing Bluetooth [4], and RFID (Radio Frequency Identification) [5] chips in the building. Although the aforementioned technology provides very accurate location information, it often requires extra time and high cost for hardware installations. Moreover, the localization systems cannot work properly and in some situations entire system goes down in the absence of the installed hardware. The latter approach relies on either the utilization of omnipresent phenomena such as geomagnetic anomalies or it uses already available off the shelf hardware. One such example is the sensors in the mobile phones such as the use of accelerometer, gyroscope, etc. The idea of using the magnetic field anomalies for indoor localization has gained wide attraction during the last few years [6,7,8]. Although this technology is prone to drift error, it is simple, cheap and does not require installation and maintenance of any additional infrastructure. Table 1 discusses the technologies used for indoor positioning and localization [9,10].

We focus on the infrastructure free approach as all smartphones have built in sensors which can easily be adopted for indoor localization. These sensors, developed using Micro-Electric Mechanical Systems (MEMS), are becoming more reliable than before. This research aims to use three such sensors: magnetometer, accelerometer and gyroscope. The contributions of this research work can be summarized as below:

1. An algorithm is presented to generate the patterns using the geomagnetic data. The generated patterns are computationally efficient to process.
2. A deep learning based artificial neural network is presented to detect user states of walking and stationary using gyroscope data.
3. An efficient infrastructure free user indoor localization algorithm is presented for when the user’s initial position is unknown.

The rest of the paper is organized in the following manner. Section 2 gives an overview of the geomagnetism, its important characteristics and fingerprinting approach. Related research work is described in Section 3. Section 4 is about the proposed method and its working mechanism. The performance of the proposed method is discussed along with the results in Section 5. Discussion is given in Section 6. Finally the conclusion is drawn in Section 7.

## 2. Geomagnetism Overview

The earth’s geomagnetic field is the natural phenomenon which extends from the earth’s interior out until it meets the solar wind. The magnitude of magnetic field at earth surface extends between 25 and 65 μ Tesla. Due to its ubiquity, it can be taken advantage of easily for indoor positioning without any infrastructural dependency. However, magnetic field measurements have certain problems that need to be addressed properly before we use it for positioning and localization.

### 2.1. Changes in Magnetic Filed Strength over Time

**Figure 1:**
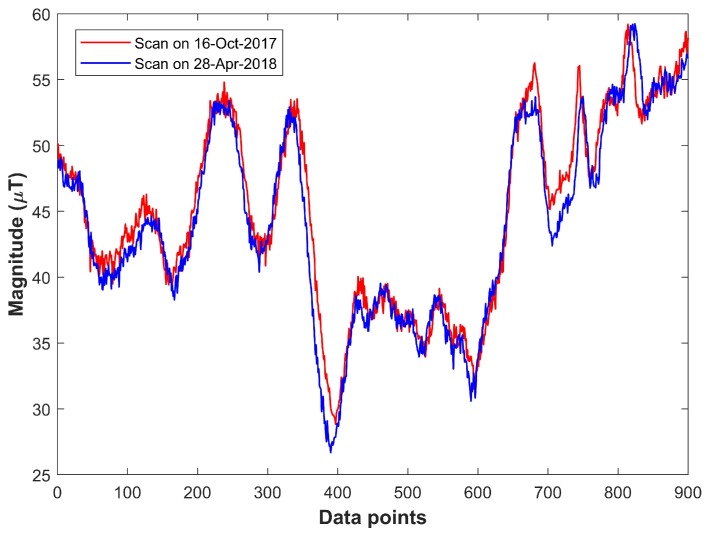
Magnetic intensity values with Galaxy S8 on different dates.

It is observed that magnetic field strength varies in space and over time [11], which is the reason the world magnetic model is designed for a period of five years. Figure 1 shows the results of collected magnetic field measurements during different periods of time. The graph shows the magnetic field strength values collected using Galaxy S8 within the duration of about five months. It clearly indicates that the magnitude of collected magnetic field strength exhibits different attitude at different time. The *x*-axis indicates the measurements generated every 100 ms, while *y*-axis shows the total magnetic field strength.

### 2.2. Device Diversity

**Figure 2:**
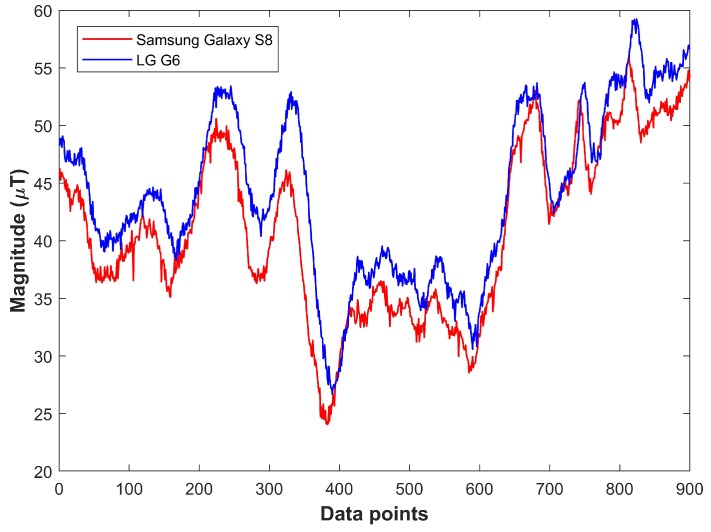
Magnetic intensity values with various smartphones for same location at same time.

Previous studies [12,13] show that various devices measure different values for the magnetic field strength of the same place, which is confirmed by our experiment. Two different phones, Samsung Galaxy S8 and LG G6, are used for the experiment. Figure 2 corroborates that different devices show different behavior along the same path at the same time.

### 2.3. Geomagnetic Fingerprinting

Fingerprinting is the process of generating and storing fingerprint signatures associated with each location index. These fingerprints are normally the averaged RSSI (Received Signal Strength Indicator) values in the case of WiFi. Fingerprinting has been utilized for localization systems during the last few decades and it comprises of two phases: the off-line phase, also called the training phase, and the on-line phase, also called the localization phase. The training phase is the survey of the buildings to collect the data for which we intend to develop localization system. Normally, several observations are collected for each point and their averaged values are stored in the database along with the unique index value of that point. Localization is performed when user sends the data of certain unknown point and that data are matched against the fingerprint database. Various matching techniques are used for the said purpose, including Euclidean distance, Manhattan distance, K nearest neighbor, etc. The process of fingerprinting is not a trivial task for a variety of reasons. First, since the fingerprint signatures are normalized values, many observations are needed to be taken for each point. Second, to achieve a good localization accuracy, the fingerprint database should be large enough. Last is the maintenance and updating of the database as any new instalment in an indoor environment requires updating the fingerprint database accordingly. Another very important factor which potentially affects the accuracy of localization system is the uniqueness of the signature. Each point should have a unique signature or the accuracy is compromised otherwise. This research aims to build a fingerprint based indoor localization system using geomagnetic sensor, accelerometer and gyroscope. We focus to resolve the last two problems mentioned above as well.

## 3. Related Work

The application of magnetic fields to solve the problem of indoor positioning and localization can be broadly categorized into two groups: active magnetic fields and passive magnetic fields. The former approach accomplishes this task by generating artificial magnetic fields. Sheinker et al. and Blankenbach & Norrdine in [14,15] utilized the same technique to get sub-meter accuracy in small testing areas. These active magnetic fields are strong and less affected by the environment, hence providing very good accuracy. The latter approach, on the other hand, benefits from earth geomagnetic field and is getting more attention from industry and academia. Since we focus on latter approach, the following section discusses few works related to this approach.

Chung et al. in [16] developed an indoor localization system using magnetic disturbances as a fingerprint. The magnetic sensor worn on the chest is used for the system. The authors compared the performance of their system with RADAR, Horus, WhereNet, Ubisense and GSM fingerprinting and showed that the system performs well in terms of precision (90% within 1.64 m) in positioning, as compared to other systems. One limitation of the system is that, in the case of larger fingerprinting map, the error goes higher. The proposed system uses chest hung sensor instead of smart phones, which makes it less vulnerable to user shaking and moving.

Li et al. in [17] investigated the use of geomagnetic field for the indoor localization using smart phone sensors. They used geomagnetic field value alone for the localization and proved that, if more elements of geomagnetic fields are used, the error is very small. However, the error may rise up to 20 m or higher in certain cases and user may be positioned in other than the original building as well if there is no knowledge of the original building. Thus, the authors suggest the use of other sensors along with geomagnetic field to remove such errors and increase precision.

Li et al. in [18] use HMR2300 and Galaxy Nexus to test the indoor localization based on geomagnetic fingerprinting. HMR2300 is placed on a trolley both to generate the database and test system localization accuracy. The results show that the error lies between 0.6 and 6.9 m without normalization. However, the accuracy is very poor in the case of using Galaxy Nexus and it goes up to 10 m. The authors suggest to use geomagnetic filed values with other positioning techniques such as WiFi to reduce the error. The author used a trolley to make the fingerprinting database; however, in real scenarios, while using mobile phone, the geomagnetic field strength values vary, which in turn can lead to higher error. Moreover, only one floor of 36 × 2 m dimension is used for the experimentation with only 20 points in total, thus the results cannot be generalized.

Zhang et al. in [19] proposed geomagnetism fingerprinting based navigation system which utilizes the crowd sourced built geomagnetic map for indoor navigation. The system includes the modules for map generation, localization and navigation using geomagnetic data. A revised Monte Carlo localization approach is used to find the indoor location. To find the initial position the system uses the continuous geomagnetic data samples of few tens of seconds. The algorithm is able to converge 90% of the errors to approximately 5 m with 30 s of data. Although the system is able to get the initial position with low error, 30 s time is very long for location estimation. Even at the velocity of 0.8 m per second, user travels approximately 24 m in the given time. Such long time may not be available in many real time situations, not to mention the fact that user frequent change of direction may make location finding more difficult as well.

Jung, J., Lee, S.M., and Myung, H., in [20] addressed the problem of robot relocation using ambient magnetic and radio measurements. They propose magnetic SLAM (Simultaneous Localization and Mapping) which is based on Rao–Blackwellized particle filter and grid-based SLAM frameworks. They make the use of two magnetometers (Honeywell, HMR 2300) and one radio range sensors (Nanotron, nanoLOC TRX) to improve the performance of SLAM. The results of the approach show a mean error of less than 1 m for both approaches. Although the results of their algorithm are quite attractive, few points are very critical for the practical implementation of their approach. First, they placed two magnetometers on robot at a specified distance from each other. The distance between the magnetometer is very important, as, if the magnetometers come close, they can interfere the geomagnetic readings, which will lead to incorrect magnetic intensities. Second, the place tested for the approach is very small, only few meters square. Third, their approach cannot be adopted for localization using smart phone, as it is not possible to deploy multiple magnetometers on a smartphone.

An indoor navigation system based on WiFi and geomagnetic fingerprinting is presented in [21]. The error of geomagnetic positioning is controlled by restricting search space with the help of WiFi APs (Access Point). The system is tested in two distinct areas with dense APs and with scattered APs. The experiment is conducted using Samsung galaxy S3 and S4. The experiment results show that the average error is reduced to 4.5 m when utilizing WiFi-aided geomagnetic navigation, which is up to 10.2 m in the case when WiFi alone is used. The results show that WiFi-aided magnetic matching positioning serves as a reliable solution for indoor navigation. This approach relies heavily on the use of WiFi to restrict the search space for geomagnetic matching and if WiFi is not used the error for geomagnetism alone goes up to 16.6 m. In addition, WiFi approach is infrastructure dependent and its failure result in system being non functional.

Meng et al. in [22] introduce an indoor localization which uses the magnetic sensor of the smartphone to localize a user. The magnetic map is formed with the help of local weight regression, as presented by Cleveland [23]. The LWR uses the local data to fit points with the help of polynomials weighted fitting and least square method is used to calculate the polynomial coefficient. The user collected magnetic data are filtered with moving average filtering model and then user’s location is estimated using a particle filter method. The experiment is performed in 27 × 7 m building with Honor7 smartphone and the results demonstrate that the average error with filtered data is 0.229 m while the unprocessed data show an error of 0.394 m. The test environment is only one building with short space and the experiment is conducted with only one smartphone, thus the device dependence is not studied. In addition, as mentioned in the paper, the measurements are taken with fixed height and smartphone direction and how the magnetic attitude will vary with changing height is not studied. An indoor localization system is offered in [24] which is based on WiFi, image and magnetic fingerprints, Bluetooth and people co-occurrence. The image database contains the images of each room which helps to narrow down the search area. The SIFT (Scale Invariant Feature Transform) is used to extract the features from the images. The user time-specific activities are also taken into account to determine the location. The experimental results reveal that the proposed system achieves an accuracy of 87.3% on average to successfully locate the user. The system is based on user’s social information along with the camera, WiFi, Bluetooth and smartphone sensors and uses the spatio-temporal co-occurrence information for localization which raises privacy concerns. In addition, the system is infrastructure dependent, as it uses the WiFi which is not pervasive.

A fingerprinting based localization scheme based on magnetic field strength and channel state information is discussed in [25]. A Line of Sight (LOS) identification algorithm is presented to narrow down the localization area. Afterwards, the channel state information is combined with magnetic field strength to formulate the fingerprint database. Then, localization is performed by matching the user scanned magnetic field information with the fingerprint database with the help of Multi-dimensional scaling k-nearest neighbor algorithm with Minkowski distance. The results show that the system is able to accurately locate a user within 1.7 m for 80% of test points. An indoor localization approach based on visual images and geomagnetism called VMag is presented in [26]. The approach utilizes the smartphone sensors and does not require additional infrastructure assistance. The data from magnetic sensor are fused with visual images for indoor localization. A neural network based method is also introduced for extracting the features from visual images. Afterwards, a context aware particle filter approach is used for tracking. The approach is able to achieve a probability of 91% for 1.34 m accuracy for four different tested environments including a laboratory, a garage, a canteen and an office.

An indoor localization system is presented in [27] which utilizes the magnetic field information to locate a user. Instead of using the magnetic intensity alone, it considers magnetic *x*, *y* and *z* values and calculates representative features. The features including entropy, power of the coefficients, variance, variance of Fast Fourier Transform, intensity, Zero Crossing Rate, kurtosis, skewness and correlation coefficient are extracted and later used for localization process. The clustering is performed as well by taking into account the magnetic intensity. A classifier is designed to estimate the location based on the extracted features. The presented approach shows good results, however with a single device.

28 presented an indoor localization approach based on camera, WiFi and inertial sensors including accelerometer, gyroscope, compass and magnetometer. Initially, the scene is identified using the camera on the smartphone and deep learning has been utilized for the said purpose. Fingerprinting approach is used for WiFi and geomagnetism to aid the localization process. The proposed system is able to achieve an accuracy of 1.32 m at 95%. Even though the system achieves a very good accuracy, it is not always practical to hold the mobile phone in a position to acquire the image of the scene which is the very pivotal component of the proposed system. In addition, using mobile phone camera consumes the phone battery very quickly as well. Moreover, the draw back of using the camera is that it cannot work in dark environment. Another fact which cannot be undermined is that the system also uses the WiFi fingerprinting which makes it infrastructure dependent.

This research presents an indoor localization system which benefits from the geomagnetic disturbances prevail in indoor environments. These disturbances are caused by ferromagnetic materials such as doors, elevators, concrete containing iron, etc. The majority of the already available indoor positioning and localization systems based on geomagnetism use fingerprinting technique. The main drawbacks of the traditional fingerprinting based techniques are that the database needs to be updated periodically otherwise the accuracy of the localization systems is affected. Secondly, the geomagnetic data are device dependent which means that measuring magnetic flux intensity using two different smart phones shows very different values. This makes it very difficult to use geomagnetism for indoor localization for all devices alike. The above mentioned problems are resolved using the geomagnetic patterns. Thus, instead of using magnetic flux intensity values, we use the patterns formed by these values. Figure 2 shows that patterns for magnetic intensity are very similar for different devices, thus we make the database of these patterns. In addition, even though the magnetic field strength values changes, they form the similar patterns. Thus, patterns are not affected by the change in the geomagnetic strength values. The proposed method is discussed in detail in the next section.

## 4. The Proposed Method

**Figure 3:**
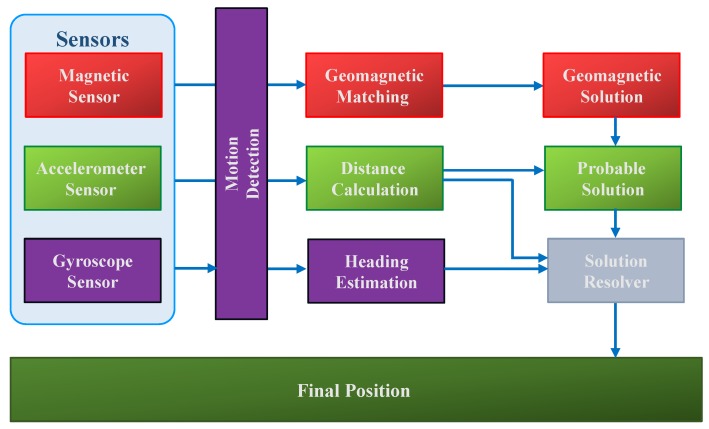
The proposed system block diagram.

The indoor localization that this study presents leverages off-the-shelf sensors already available in smartphones. Figure 3 shows the block diagram of the proposed system. We make the use of three sensors including magnetic sensor, accelerometer and gyroscope. The gyroscope is used to determine the heading estimation of the user. Additionally, it has been utilized to ascertain the state of the user i.e., whether he is moving or stationary. The accelerometer and magnetometer are employed to estimate the user current location. Although, traditionally, accelerometer needs the initial position to determine the next position, we use it without the initial position in this case. The magnetometer is the main sensor which computes the user location by taking the advantage of the accelerometer. Our proposed system incorporates three modules and, in the following section, each module is discussed separately.

### 4.1. The Gyroscope Module

In the current study, the purpose of using the gyroscope is twofold: motion detection and the heading estimation. Even though accelerometer is used traditionally for the motion detection, we use gyroscope for the same purpose for two reasons. First, the gyroscope supports fast data rate than accelerometer so working at higher data rate is possible. Second, gyroscope is more tolerant to noise, so, with a little preprocessing, data quality is good to be used for the system. Various techniques exist that can be adopted for motion detection including Artificial Neural Networks (ANN), Support Vector Machines (SVM), Decision Trees (DT), Principal Component Analysis (PCA), etc. We use ANN in our work, as it is easy to implement and shows good performance as well.

**Figure 4:**
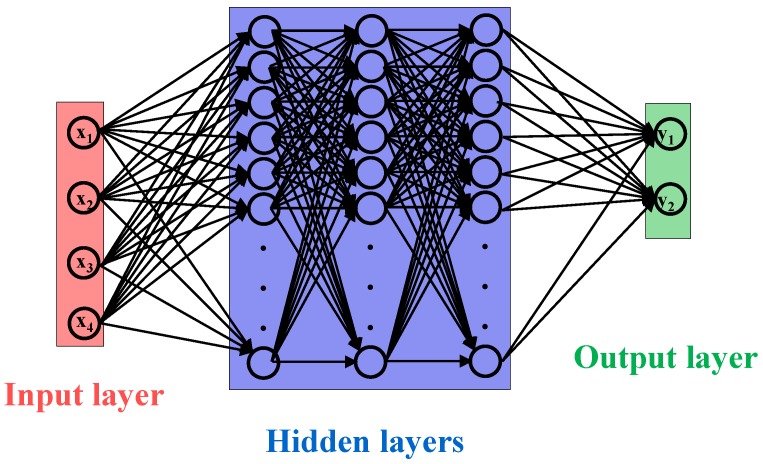
The architecture of an ANN model.

**Figure 5:**
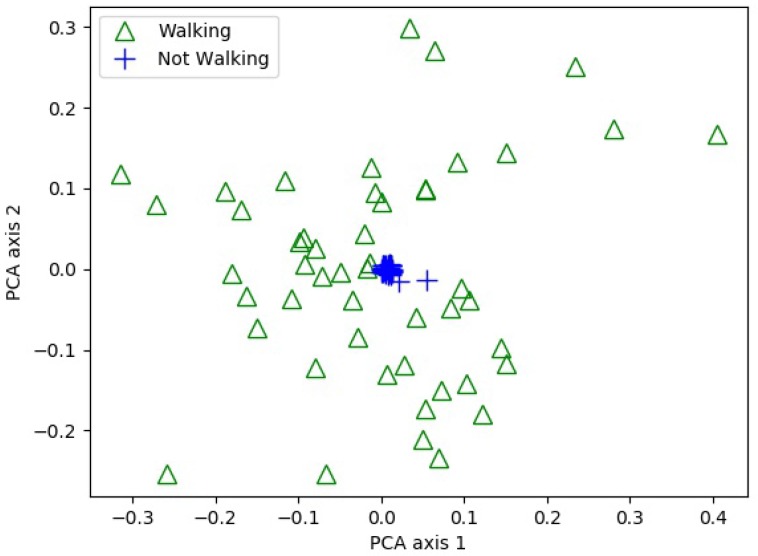
Principal component analysis of features used for motion detection.

The ANN is the mathematical model based on the human brain neuron and it works well for pattern recognition, forecasting, data compression, etc. A typical ANN is consisted of an input layer, one or more hidden layers and an output layer each containing small units called neurons or nodes, as shown in Figure 4. The layers are interconnected with the directed edges and each edge carries a weight which is used to determine the activation of the neuron in the next hidden layer with the help of a mathematical function. Similarly, another function computes the output of the ANN. ANNs are used to determine the decision boundaries in the form of weights and biases, which is achieved with the help of training. ANNs are characterized into supervised and unsupervised based on their training methods. The number of the input nodes depend on the number of the features and are selected equal to the number of features. The feature selection is the most important process in the ANN modeling. Integrating too many features degrades the performance of the ANN model; similarly, the features which have no or less variance produce poor results. Conventionally a feature analysis algorithm such as PCA is utilized to analyze the behavior of the extracted features. A PCA is performed in the current study to analyze how well the selected features can classify the motion and shown in Figure 5.

The weights of the ANN are determined using the back-propagation algorithm [29] in which outputs are computed and then errors are propagated backwards to adjust the weights accordingly. This process is repeated until the error is converged to a certain threshold. In the current study, an ANN with five hidden layers and five nodes is used with supervised training. The priority of using ANN over other traditional methods is set for motion detection in current study because the use of ANN is reported in [30] for motion classification, in [31] for motion classification using smartphone, in [32] for step counting based on accelerometer and gyroscope data, in [33] for step detection and step length estimation, and in [34] for stride detection using smartphone.

The input vector $X_{t}=\left\{x_{0},x_{1},\ldots ,x_{m}\right\}$ is used for the ANN where *t* shows the time and *m* is the size of the input vector. For the current study, *m* is five because we use four features from the gyroscope and a bias node. The details of the features used as an input vector are given in Table 2.

The output vector $Y_{t}=\left\{y_{0},y_{1},\ldots ,y_{n}\right\}$ is used for the ANN where *n* is the size of the output vector, which is two in our study. We use the gyroscope data to determine whether the use is walking or stationary at a particular time. We use sigmoid activation function for the ANN.

(1) $$\sigma {\left(Z\right)}_{j}=\frac{e^{Z_{j}}}{\Sigma_{k=1}^{K}e^{Z_{k}}}$$

The sensor data from smartphone sensors is collected at a sampling frequency of 10 Hz and is pre-processed using a low pass filter to reduce the noise effect. The selected features value varies significantly when the user changes its state from stationary to walking. For training the ANN, 5000 samples of every feature are used. The ANN for user state prediction achieves an accuracy of 95%.

### 4.2. The Acceleration Module

The accelerometer is used to calculate the distance traveled by the user in a given time frame *f* where the size of *f* for this study is 1 s. The accelerometer provides three-axis acceleration values and the total acceleration is calculated as follows:

(2) $$a=\sqrt{a_{x}^{2}+a_{y}^{2}+a_{z}^{2}}$$

We use step detection model to calculate the distance for each frame. A step detection algorithm is proposed as well which works on the acceleration data. The algorithm has the following steps:

(1) Compute the magnitude of the acceleration for a given sample *i*
  (3) $$a_{i}=\sqrt{a_{x_{i}}^{2}+a_{y_{i}}^{2}+a_{z_{i}}^{2}}$$
(2) Compute the variance in acceleration using
  (4) $$\sigma_{i}=a_{i}-\mu_{a}$$
  where $\mu_{a}$ is empirically calculated value for medium walking speed and $\mu_{a}=\Sigma_{i=1}^{n}\frac{1}{n}$.
(3) Find the local maxima as step candidates and then apply Threshold 1 and Threshold 2. Threshold 1 $\Sigma_{i=1}^{n}\frac{1}{\left(\frac{3}{f}+1\right)}$ is applied on acceleration and Threshold 2 $\left\lfloor \mu_{d_{LM}}\right\rfloor$ is on the distance between the peaks.

After step detection, we use Weinberg model [35] to measure the step length:

(5) $$stepLength=k\sqrt[{4}]{a_{max}-a_{min}}$$

where $a_{max}$ and $a_{min}$ represent the maximum and minimum values of acceleration for the given frame and k is empirical step length estimation. The value of k is 0.55 in the original article but we use a value of 0.52 as it gives more accurate results for our data.

When step detection and step length estimation is complete, we can calculate the position of the user using the following equation:

(6) $$\begin{array}{c} Pos_{x_{i+1}}=x_{i}+stepLength*\cos\left(\psi_{i}\right),\\ Pos_{y_{i+1}}=y_{i}+stepLength*\sin\left(\psi_{i}\right) \end{array}$$

where ψ represents the heading angle (yaw) and we make the use of Euler angles to calculate it as:

(7) $$\begin{array}{c} \left[\begin{array}{c} \phi_{roll}\\ \theta_{pitch}\\ \psi_{yaw} \end{array}\right]=\left[\begin{array}{ccc} 1 & sin\phi tan\theta & cos\phi tan\theta \\ 0 & cos\phi & -sin\phi \\ 0 & sin\phi sec\theta & cos\phi sec\theta \end{array}\right]\left[\begin{array}{c} gyro_{x}\\ gyro_{y}\\ gyro_{z} \end{array}\right] \end{array}$$

The calculated distance using accelerometer module is used to assist the magnetometer module to find the exact position of the user. The details of this process are discussed in the next section.

### 4.3. The Magnetometer Module

**Figure 6:**
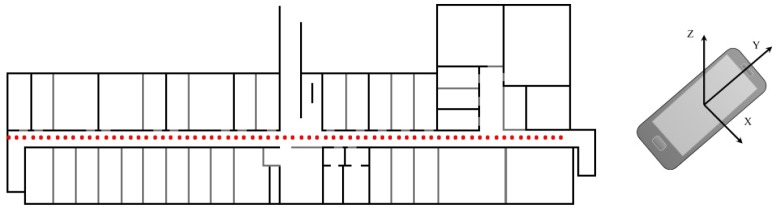
Fingerprinting points in IT building (**left**); and magnetometer axes on the smartphone (**right**).

The magnetometer module is the key part of the system which decides the user location in the indoor environment. The location of the user is found by matching the user scan with the fingerprint database. The database contains patterns formed by magnet *x*, magnet *y* and magnetic *z* values for each point. Our experiments reveal that accuracy is higher if *x*, *y* and *z* magnetic values are matched as compared to magnetic field intensity B alone, so we store the patterns formed by *x*, *y* and *z* magnetic values. Since the orientation of the smart phone is fixed in front of the user, as shown in Figure 6, the values of three magnetic axis can be used. Additionally, the fixed behavior of smartphone orientation guarantees the quality of data as well and sensor and shaking noise is removed by using a low pass filter. For fingerprint database, it is not appropriate to take continuous scans and average them as various scans may have different lengths of data due to the varying speed of the user. Instead, we take 100 observations at each point separated by 1 m and then these observations are averaged. Afterwards, interpolation is used to generate data values between these points.

The fingerprint database approach has associated disadvantage of its need to be updated periodically. Moreover, various smartphones exhibit different geomagnetic values at the same time for the same place which makes it impossible to use the fingerprint approach for all smartphones. Preferably, we use the patterns formed by smartphones as compared to the geomagnetic values themselves. Pattern matching in itself is a computationally expensive process which may hinder the performance of the system, so we present the concept of Binary Grid (BG) which stores the patterns in a binary form. We call it binary as it contains only two integers: 0 and 1. The 1 is where we have a magnetic value and 0 otherwise. Algorithm 1 is proposed to generate the BG for given geomagnetic intensity values. It is easy and computationally efficient to convert the values to patterns and store and extract them. Here, we explain the algorithm with the help of a toy example.
**Algorithm 1** Generate geomagnetic pattern1:$max_{v}=max\left(G_{w}\right);$2:$min_{v}=min\left(G_{w}\right);$3:M⟵length(f);4:$N⟵round(max_{v}-min_{v})+1;$5:$\mathrm{Define}\;B_{m}\;\mathrm{of}\;\mathrm{MxN}\;\mathrm{order};$6:**for**j⟵toM**do**7:    $r⟵round(max_{v}-G_{w}\left(j\right))+1;$8:    $B_{m}\left(r,j\right)⟵1;$ 9:**end for**


**Figure 7:**
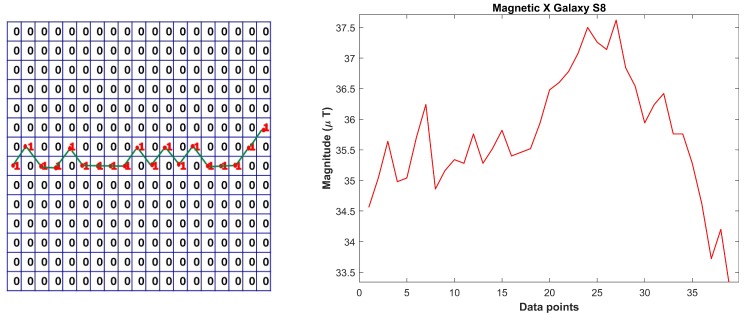
Sample binary grid for first 20 data points (**left**); and magnetic field data (**right**).

Let us say that we have to transform the geomagnetic values shown in Figure 7 to the pattern and store it in the BG. First, we need to decide the size of the BG. The total number of rows in the grid is the difference of maximum and minimum magnetic values, while the columns are the length of the frame or, in this case, the total data points taken.

**Figure 8:**
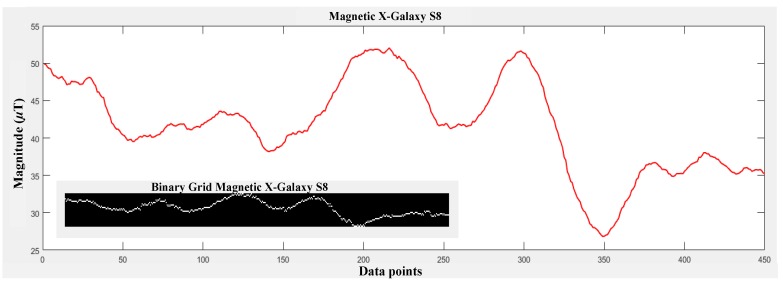
Geomagnetic pattern and resultant binary grid.

First, a matrix containing zeros of *M* × *N* size is declared where *M* is number of rows and *N* is the number of columns. Then, each magnetic data point is taken and converted to a row number by subtracting it from the maximum data point and rounding it. Afterwards, a 1 is added to the calculated row and this process is repeated for all the data values. The BG shown in Figure 7 (left) is for the first twenty values only of the graph shown in Figure 7 (right), while Figure 8 shows the magnetic *x* axis for Galaxy S8 and its associated BG.

For the current study, magnetic data values from Samsung Galaxy S8 are gathered to make the BG. Afterwards, this grid is used to match the user scans for both Samsung Galaxy S8 and LG G6. We use a 1 s frame at a sampling rate of 10 Hz to perform the pattern matching. Each frame is taken, converted to the pattern using the given algorithm and then matched against the BG (geomagnetic pattern database). The point with the lowest error is selected as the user location and error is calculated using the Euclidean distance. Algorithm 2 is used as the localization algorithm. Table 3 shows the notations used in the algorithm.
**Algorithm 2** User positioning using geomagnetic and acceleration data.1:**for**i⟵to5**do**2:    **for**
j⟵toW
**do**3:        $D_{a}\left(j\right)⟵calculateDistance\left(A_{w}\right);$4:         $S\left(j\right)⟵generatePattern\left(G_{w}\right);$ 5:        $P_{g}\left(j\right)⟵findGeomagneticPosition\left(S\left(j\right)\right);$ 6:    **end for** 7:    $P_{c}⟵generatePositionCandidates\left(P_{g},D_{a}\right);$ 8:    $P_{i}⟵getPosition\left(P_{c},P_{g}\right);$ 9:**end for**10:$P_{f}⟵finalizePosition\left(P,D_{a}\right)$;


First, a user sample of geomagnetic values is taken and converted to a pattern using the given algorithm. Then, that sample pattern is matched against the pattern database. The point with the lowest Euclidean distance is taken as the geomagnetic position. This process is repeated for ten consecutive samples.

**Figure 9:**
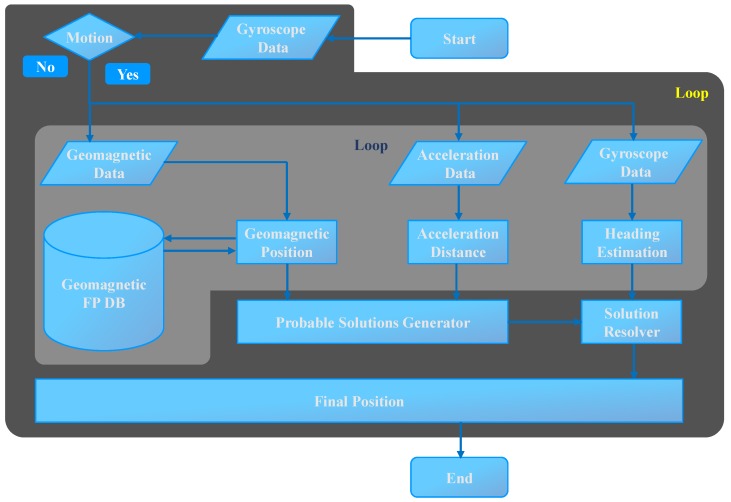
Geomagnetic positioning algorithm process.

The distance traveled by the user is also calculated using the acceleration data. Five such iterations are made and then the estimated positions using geomagnetic pattern matching for five iterations are considered for final location. The underlying reason for taking five windows is to reduce the error in matching caused by the similarity of magnetic values at different places. Even though that pattern matching performs very well in finding the user position, the user position is often miscalculated, which results in high errors. Each window is exactly one frame ahead of the previous window. This difference is used to infer the next location of the user. By taking user position for five consecutive windows, we are able to find the outliers and replace them with correct positions using the distance calculated using the accelerometer data. Since each window is separated by one frame and the average distance traveled by user in one frame is 0.85 to 0.95 m, the location calculated by the algorithm for each window should match the same, otherwise it is not correct. We follow the same logic to calculate the final location of the user. Figure 9 shows the flow chart of the proposed algorithm. In the following a toy example is given to better explain how the algorithm works.

**Toy example**: Let us say that we have user taken geomagnetic values given in Figure 8; the blue bars on the figure show the size of the frame taken for pattern matching. After the first iteration of the inner loop given in Algorithm 2, we have ten geomagnetic positions and ten distance values for the same frames. Now, we assume that each geomagnetic position is a correct estimate and adjust its preceding and conceding positions with the help of the acceleration distance we have. For example, for $P_{g}=\left\{P_{g_{1}},P_{g_{2}},\ldots ,P_{g_{n}}\right\}$ set of geomagnetic position estimates and $D_{a}=\left\{D_{a_{1}},D_{a_{2}},\ldots ,D_{a_{n}}\right\}$ set of distance data, the set of candidate positions is calculated as:

(8) $$P_{c_{n+1}}=P_{n}+D_{n+i}$$

(9) $$P_{c_{n-1}}=P_{n}-D_{n-i}$$

**Figure 10:**
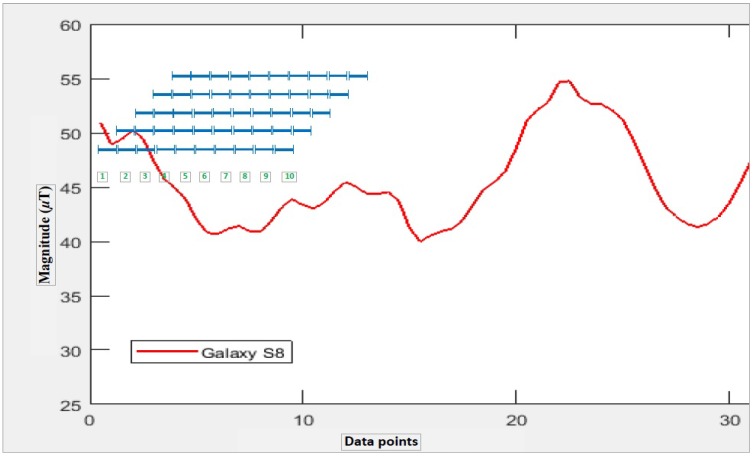
Geomagnetic positioning algorithm process.

For the data values given in Figure 10, the geomagnetic estimates and distance data are given in Table 4.

For simplicity, in this explanation, we only take x coordinate of geomagnetic estimate. The acceleration distance is in meters and show the distance for one frame. Thus, if we consider that the first geomagnetic estimate is correct, then the next positions can be calculated using the distance data and they will be 4.42, 5.25, 6.04, 6.97, 7.82, 8.72, 9.51, 10.39 and 11.2, respectively. We call these calculated positions as position candidates and we calculate all the possible candidates by considering each of these 10 geomagnetic estimates as the correct position. This yields ten sets of position candidates. The calculated candidate sets are then compared with geomagnetic estimates and positions are:

(10) $$P=\sqrt{\Sigma_{i=1}^{n}{\left(P_{gi}-P_{ci}\right)}^{2}}$$

One set of position candidates is selected as the geomagnetic positions and its first position is the starting position of the user. This process is repeated for five windows. The size of the window is same, i.e., ten frames. The only difference is that the second window is one frame ahead of the first frame and so on, as shown in Figure 10. Thus, after the five windows are finished processing, we get five starting positions. Sometimes, the calculated five positions are not consistent so we need to look for any outliers. This will ensure more stable and accurate position. We use the following equation to find the outliers.

(11) $$V=|\frac{1}{n}\left(\Sigma_{i=1}^{n}\right)x_{i}-x_{i}|$$

(12) $$O=\left\{\begin{array}{c} 1,\;ifV>\tau \\ 0,\;otherwise \end{array}\right.$$

where τ is set to 10, as we have at most 10 m data. The outliers are removed and positions are calculated using the correct positions and distance data. For example, the *x* coordinate of five positions calculated for the above given example are 3.51, 4.46, 32.46, 6.88 and 7.76, respectively. Thus, using the outlier finding equation, the third position in this sequence is an outlier and it is replaced with the correct position using the distance data. The positions corrected after the removal of outliers are called the finalized positions.

## 5. Performance Evaluation of Proposed Algorithm

### 5.1. The Experiment Setup

**Figure 11:**
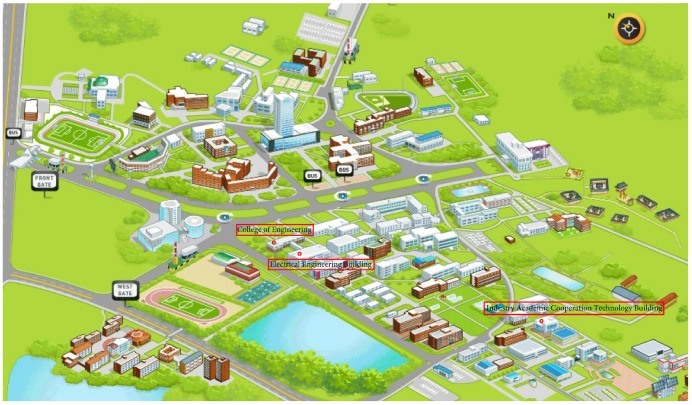
Locations of the test buildings on the map.

The experiment was performed using three different buildings located in Yeungnam University, Republic of Korea. Figure 11 shows the location of buildings used for the experiment on University map.

**Figure 12:**
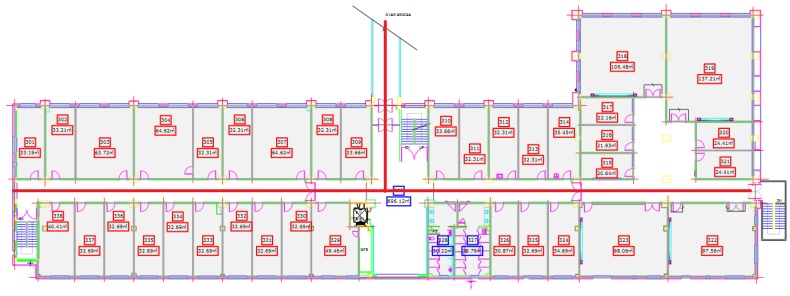
The test path on IT building floor 3.

Figure 12 shows the map of Information Technology (IT) building Floor 3. The experiment was performed in its corridor which is 90 m long and 3 m wide. Besides, it also has 25 m long over bridge connected at its mid-point. The red line on the map shows the path used for the experiment.

**Figure 13:**
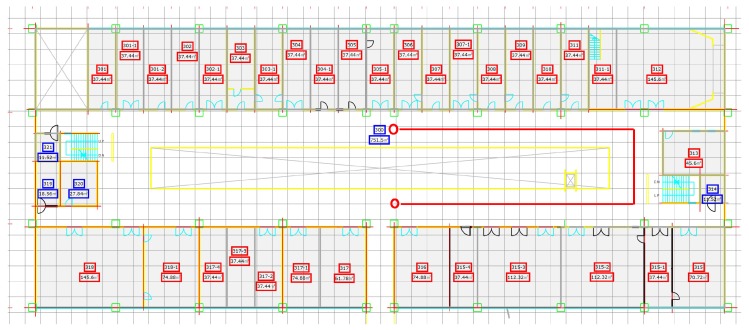
The test path on EE building floor 3.

Similarly, the third floor of the Electrical Engineering (EE) department was tested using the proposed algorithm. We tested with a different path for EE building and use a semi square path. Figure 13 shows the map of EE building and the path used for the experiment. The circles show the starting and shows the ending point of the test while the direction is not fixed and user can move to any direction. The EE building path test is 40 m long each on both sides while the other side is 15 m long.

**Figure 14:**
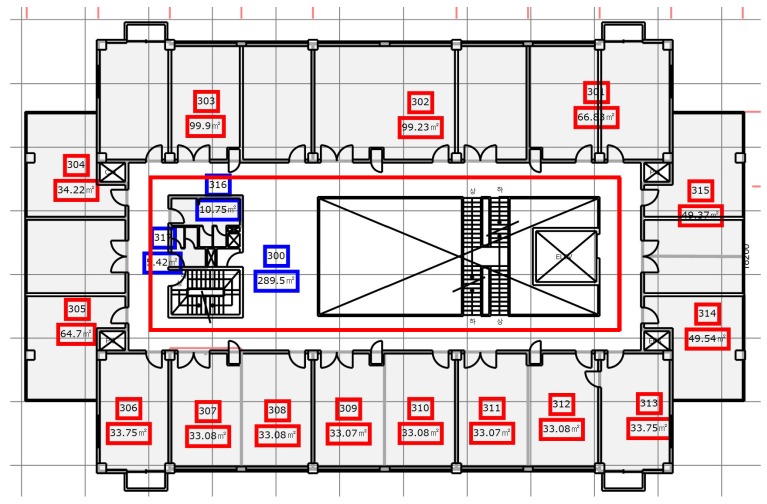
The test path on IACT building Floor 2.

The third building where the experiment was performed is Industry Academic Cooperation Technology Building (IACT) Floor 2. The path for IACT is a squared path where the ending point is from where we start the experiment. Figure 14 shows the map of IACT building Floor 2. The tested path’s dimensions are 25 × 10 m.

**Figure 15:**
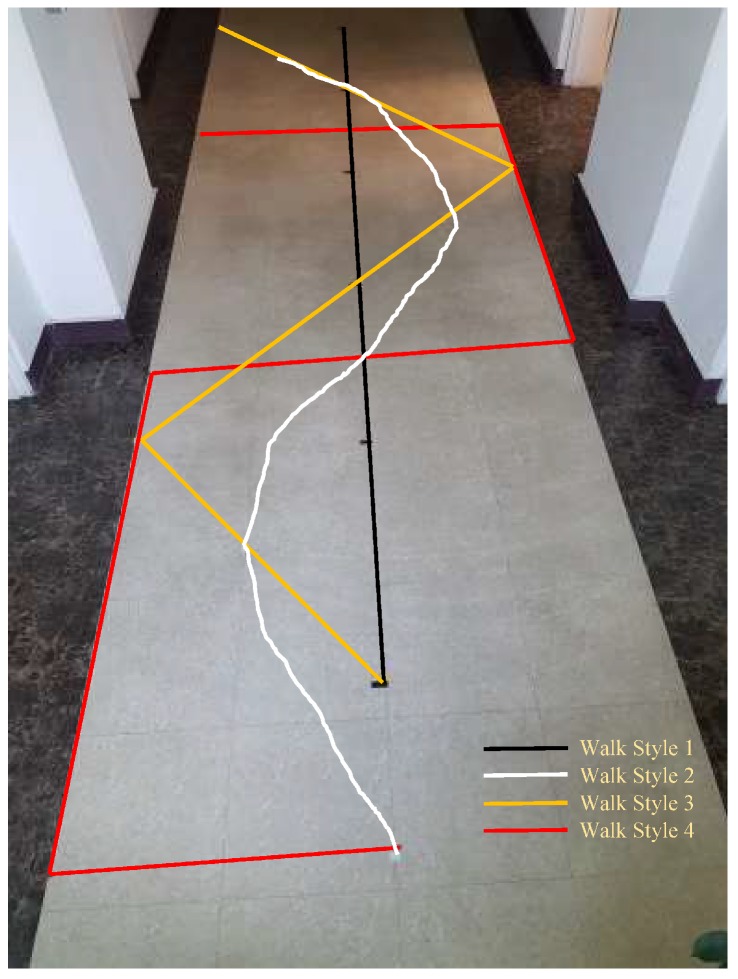
The walking styles used for experiment.

To check the performance of the system in more realistic and complicated environments, we adopted four different walking styles, as shown in Figure 15. The first walking style is to follow the straight path, as shown in Figure 12, Figure 13 and Figure 14, and it includes few turns. The second walking style is a semi circular path where the user follows a zigzag path while the third walking style is a triangular shaped path where the user walks across the straight line path at specified points. The last walking style is more complex than the previous three and it includes many turns along side the path at specified marked points. The points on the floor show the ground truth values and are used to evaluate the accuracy of localization.

### 5.2. Results

**Figure 16:**
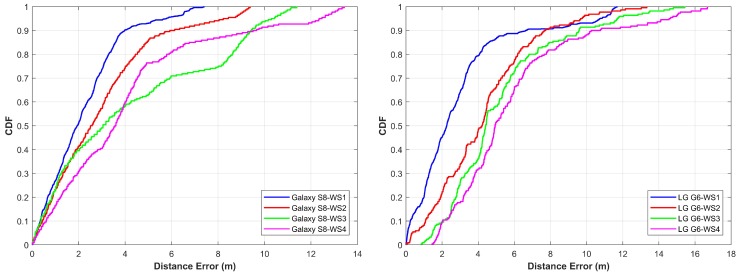
The cumulative distribution function (CDF) of Galaxy S8 and LG G6 for IT building.

In this part, we present the accuracy results of the proposed algorithm for the three buildings. Figure 16 shows the results for IT building Floor 3. The proposed algorithm is able to achieve almost similar accuracy results for both Samsung Galaxy S8 and LG G6, although both devices behave very different when magnetic intensity values are gathered. However, since we use patterns formed by the magnetic values instead of magnetic values, algorithm predicts accurate locations in both cases. To calculate the location, each point is taken without any prior knowledge of initial position and then the current location is calculated using the algorithm. The graph shown in Figure 16 is for at least 1200 calculated locations or more. Every time we start the observation from an unknown point and predict the user location with the given system. With the proposed algorithm, 50% of the time the error is 2 m for Samsung Galaxy S8 while in the case of LG G6 it is 2.3 m. The mean error is 2.16 m and 2.96 m for Galaxy S8 and LG G6, respectively. The maximum error in the case of Galaxy S8 is below 8 m, however, with LG G6, it goes up to 11.69 m for the first walking style.

**Figure 17:**
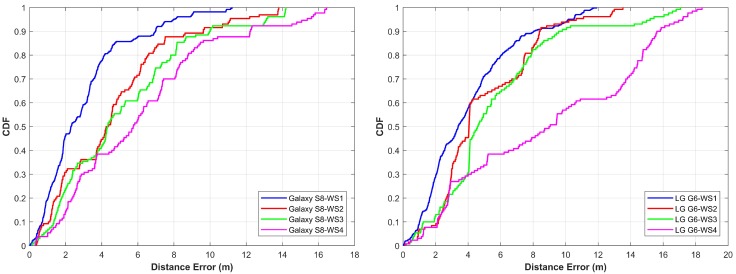
The cumulative distribution function (CDF) of Galaxy S8 and LG G6 for EE building.

Figure 17 shows the results for EE building Floor 3. The mean, minimum and maximum errors are a little higher than that of IT building. The primary reason for higher error is the nature of the path selected for the experiment. Many places for this building have very similar patterns on both sides of the path. Thus, even though the algorithm performs very well, the average error is higher in this case. Since the accuracy depends much on the uniqueness of the signature (pattern in this case), having similar patterns for the different places is causing higher errors.

The error for Galaxy S8 is 2.3 m 50% of the time while LG G6 has 3.3 m error. The maximum error for Galaxy S8 and LG G6 is now almost similar which is 11.22 m and 11.89 m respectively.

**Figure 18:**
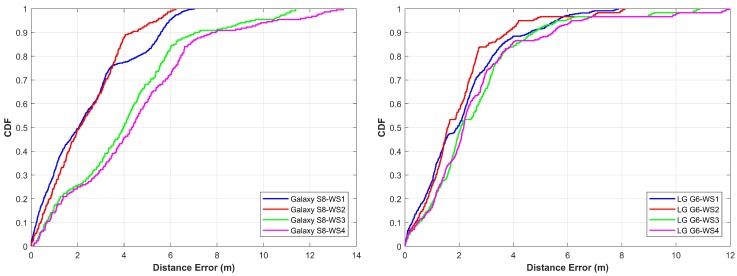
The cumulative distribution function (CDF) of Galaxy S8 and LG G6 for IACT building.

The results for IACT building Floor 3, as shown in Figure 18, are better than EE building. The error for Samsung Galaxy S8 is 2.05 m 50% of the time while it is 1.94 m for LG G6. The mean and maximum errors are also less in this case than that of the previous case. The maximum errors are 7.02 m and 7.88 m for Galaxy S8 and LG G6, respectively. The performance of the proposed method lies in three underlying factors. First is the size of the window that is used for the experimentation. It is possible to get incorrect locations even with pattern matching if we use few frames of magnetic data, however, the use of taking more frames ensures more consistent location results. Second, on many occasions, we get very dissimilar results for matching more than one frames pattern against pattern database but the technique of generating the possible localization candidates secures the closest localization points to the ground truth. The last is the use of more than one windows which are intentionally separated by exactly one frame. Since we know the average distance traveled by the user in one frame, we use it to infer the next correct location and remove the outliers.

As shown in the Figure 16, Figure 17 and Figure 18, the straight walk has high accuracy as compared to other walking styles. However, when we use a more complicated walk, the error increases. The accuracy for the first, second and third walking styles is less affected as against the walking style four where the performance is highly degraded. The underlying reason is that the magnetic field values change highly when we change the direction of the magnetometer and so does the patterns that we form from these values. Thus, although we follow a zigzag path when we follow walk two and three, the direction of the smartphone is less affected as compared to the walk four. However, with walk four, we have many turns which change the magnetic patterns resultantly. Such frequent changes in magnetic patterns results in poor pattern matching outcomes which ultimately leads to higher localization errors. Even though the algorithm performs well, walking style four is complicated, as even in real life scenarios we do not follow such paths. The statistics for all buildings are summarized in Table 5 for both devices.

## 6. Discussion

This study presents an indoor localization system based on off-the-shelf smartphone geomagnetic sensor which is further assisted by accelerometer and gyroscope sensors. The fingerprinting approach is used for localization. However, unlike the traditional fingerprint approach where intensity values are stored, a fingerprint database of intensity data patterns is built. A pattern making algorithm is presented for fast transformation of data to patterns and computationally efficient for storing and retrieving of the patterns. In addition, deep learning method is adopted to identify user walking and stationary states using gyroscope sensor. The accuracy of the deep learning ANN is 95%. The experimental results illustrate that the mean error of the proposed system is 2–3 m for different buildings. It is apparent that the accuracy depends on the uniqueness of the pattern formed by geomagnetic values and errors rise when the pattern is similar for various locations. The proposed system has advantages over other solutions as it does not need any additional hardware. Furthermore, it does not rely on any infrastructure and works well without WiFi. Another important point to mention here is that the algorithm works without any knowledge of the initial position which means that the algorithm can predict the user position just using a few frames of user data. Even though the search space is big in some cases and there is no sensor to constrain the search space, the algorithm performs very well.

## 7. Conclusions

This research proposes a system which works on smartphone built-in sensors to locate a pedestrian indoor. The system incorporates the data from three sensors, i.e., the accelerometer, magnetometer and gyroscope, for localization. The algorithm does not need any initial position to calculate the user current position. The algorithm is able to determine user position with 4 m accuracy at 75%. To evaluate the performance of the proposed system, four different walking styles are tested starting from a straight walk to more complex walk with turn through a semicircular walk. Additionally, the algorithm solves the problem of device heterogeneity as it has been tested with Samsung Galaxy S8 and LG G6 and it works in a similar fashion for both devices. Currently, it works with a fixed device behavior where the device is held in front of the user. In the future, we intend to work with varying behaviors of user for holding the device in various manners such as listening to phone call, walking with swinging device in hand, etc.

## Figures and Tables

**Table 1:** Indoor localization techniques.

Technology	Category	Error	Cost *
Infrared	Infrastructure dependent	0.5–3 m	Moderate
Acoustic signal	Infrastructure dependent	Below 1 m	Moderate
Radio Frequency ID (RFID)	Infrastructure dependent	1–3 m	Moderate
Ultra-Wide-Band (UWB)	Infrastructure dependent	Below 1 m	High
Pedestrian Dead Reckoning (PDR)	Infrastructure free	2–5 m	No
WiFi	Infrastructure dependent	2–5 m	Low
iBeacon	Infrastructure dependent	1–5 m	Low
Vision (smartphone)	Infrastructure free	Up to 5 m	No
Visible light	Semi-Infrastructure dependent	Up to 8 m	No
Magnetic systems	Infrastructure free	1–10 m	No

* The cost column shows the cost in terms of hardware required to be installed indoors and does not include any computational cost.

**Table 2:** Features used in the ANN for user state detection.

Feature	Description
varAngV	Variance in angular velocity
varAngX	Variance in angular velocity of *x*-axis
varAngY	Variance in angular velocity of *y*-axis
varAngZ	Variance in angular velocity of *z*-axis

**Table 3:** List of notations and their description used in the algorithm.

Notation	Description
A	Set of acceleration data
$D_{a}$	Set of distance calculated using acceleration data
G	Set of geomagnetic data
P	Set of positions
$P_{c}$	Set of candidate positions
$P_{g}$	Set of geomagnetic positions
$P_{f}$	Set of finalized positions

**Table 4:** Geomagnetic positions and associated distance.

**Geomagnetic estimate**	3.51	7.91	10.8	50.4	40.9	8.93	20.7	70.1	30.9	2.35
**Acceleration distance**	0.87	0.91	0.83	0.79	0.93	0.85	0.90	0.79	0.88	0.81

**Table 5:** The summary of the results with both devices for all buildings.

Building	Device	Minimum	Maximum	Mean	50% Error	75% Error
IT floor 3	S8-WS1	0.00	7.41	1.93	1.93	3.01
	S8-WS2	0.00	9.38	2.92	2.50	4.01
	S8-WS3	0.00	11.38	4.17	3.05	5.91
	S8-WS4	0.04	13.43	4.07	3.52	4.82
	G6-WS1	0.00	11.69	2.96	2.26	3.40
	G6-WS2	0.00	13.30	4.30	4.20	5.75
	G6-WS3	0.85	15.43	5.17	4.40	6.18
	G6-WS4	1.45	16.69	5.84	4.90	6.72
EE floor 3	S8-WS1	0.00	11.22	2.98	2.31	3.72
	S8-WS2	0.37	13.79	4.60	4.25	6.17
	S8-WS3	0.13	14.19	5.13	4.30	7.25
	S8-WS4	0.32	16.44	6.11	5.74	8.28
	G6-WS1	0.01	11.89	3.99	3.30	5.50
	G6-WS2	0.10	13.52	4.92	4.05	7.40
	G6-WS3	0.20	17.06	5.60	4.55	7.41
	G6-WS4	0.16	18.39	8.90	8.97	14.38
IACT floor 2	S8-WS1	0.00	7.02	2.42	2.05	3.38
	S8-WS2	0.01	6.22	2.31	2.08	3.44
	S8-WS3	0.08	11.38	4.02	3.96	5.52
	S8-WS4	0.08	13.43	4.45	4.30	6.12
	G6-WS1	0.00	7.88	2.12	1.94	2.97
	G6-WS2	0.00	8.11	1.96	1.60	2.51
	G6-WS3	0.05	10.87	2.56	2.05	3.15
	G6-WS4	0.07	11.96	2.61	2.17	3.30
